# Suppressor of Ty homolog-5, a novel tumor-specific human telomerase reverse transcriptase promoter-binding protein and activator in colon cancer cells

**DOI:** 10.18632/oncotarget.5301

**Published:** 2015-09-25

**Authors:** Rui Chen, Jing Zhu, Yong Dong, Chao He, Xiaotong Hu

**Affiliations:** ^1^ Department of Colorectal Surgery, Sir Run Run Shaw Hospital, Zhejiang University, Hangzhou 310016, China; ^2^ Biomedical Research Center and Key Laboratory of Biotherapy of Zhejiang Province, Sir Run Run Shaw Hospital, Zhejiang University, Hangzhou 310016, China

**Keywords:** SPT5, SUPT5H, promoter, telomerase activity, colon cancer

## Abstract

The human telomerase reverse transcriptase (*hTERT*) promoter promotes differential *hTERT* gene expression in tumor cells and normal cells. However, information on the mechanisms underlying the differential *hTERT* transcription and induction of telomerase activity in tumor cells is limited. In the present study, suppressor of Ty homolog-5 (SPT5), a protein encoded by the *SUPT5H* gene, was identified as a novel tumor-specific *hTERT* promoter-binding protein and activator in colon cancer cells. We verified the tumor-specific binding activity of SPT5 to the *hTERT* promoter *in vitro* and *in vivo* and detected high expression levels of *SUPT5H* in colorectal cancer cell lines and primary human colorectal cancer tissues. *SUPT5H* was more highly expressed in colorectal cancer cases with distant metastasis than in cases without distant metastasis. Inhibition of endogenous *SUPT5H* expression by *SUPT5H* gene-specific short hairpin RNAs effectively attenuated *hTERT* promoter-driven green fluorescent protein (GFP) expression, whereas no detectable effects on CMV promoter-driven GFP expression in the same cells were observed. In addition, inhibition of *SUPT5H* expression not only effectively repressed telomerase activity, accelerated telomere shortening, and promoted cell senescence in colon cancer cells, but also suppressed cancer cell growth and migration. Our results demonstrated that SPT5 contributes to the up-regulation of *hTERT* expression and tumor development, and *SUPT5H* may potentially be used as a novel tumor biomarker and/or cancer therapeutic target.

## INTRODUCTION

The human telomerase holoenzyme comprises a telomerase reverse transcriptase (hTERT), telomerase RNA component, and telomerase accessory proteins [1, 2]. As the core catalytic subunit of telomerase holoenzyme, hTERT synthesizes telomeric DNA repeats onto the ends of chromosomes to maintain chromosomal integrity and stability [3]. With the exception of stem and germ cells and other highly proliferating cells, telomerase is continuously repressed in most normal somatic tissues. In contrast, 70%–90% of cancer cells stably express this enzyme, which is reactivated during malignant transformation [4, 5]. In human cancer, expression of telomerase is positively correlated with tumor aggressiveness and metastatic potential [6–10]. There is a striking correlation between the presence of *hTERT* mRNA and telomerase activity [11], suggesting that *hTERT* expression is regulated by changes in the rate of transcription. Diverse transcription factors critically control the process of its transcription. Lack of *hTERT* expression in normal cells may be due to one or more repressors. Several transcription factors, including oncogene products (e.g., c-Myc) and tumor suppressor gene products (e.g., WT1 and p53), control *hTERT* transcription when overexpressed [12], although its role in inducing *hTERT* activation during carcinogenesis remains elusive.

We speculate that some transcription factors existing in human colon cancer cells differentially or specifically bind to the *hTERT* promoter to facilitate *hTERT* expression and promote tumorigenesis and development. The present study provides evidence that the suppressor of Ty homolog-5 (SPT5), a protein encoded by the *SUPT5H* gene, is a novel tumor-specific *hTERT* promoter-binding protein in colon cancer cells. Our results demonstrate that SPT5 contributes to the upregulation of *hTERT* expression and tumor development, and *SUPT5H* maybe a novel tumor biomarker or a cancer therapeutic target.

## RESULTS

### Detection and identification of tumor-specific *hTERT* promoter-binding proteins

**Figure 1:**
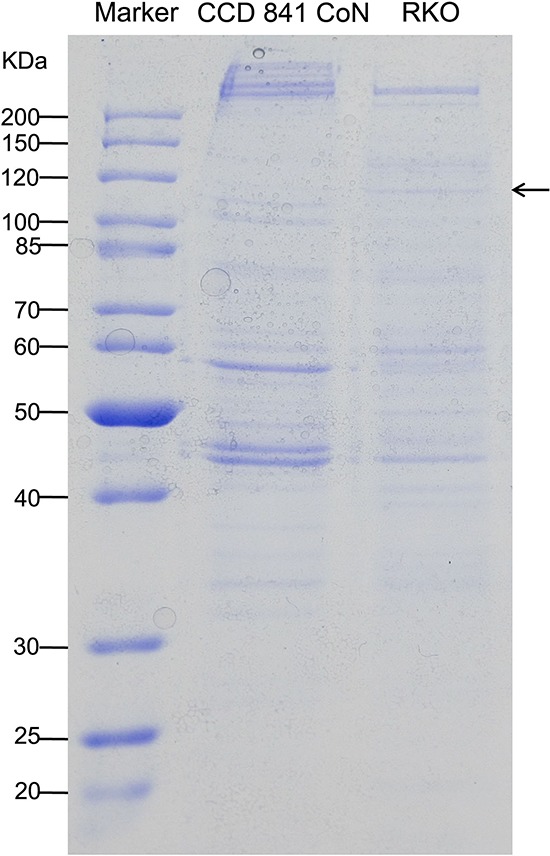
Detection and identification of tumor-specific hTERT promoter-binding proteins Nuclear proteins were extracted from normal colon epithelial cell line (CCD 841 CoN) and colon cancer cell line (RKO), respectively. The *hTERT* promoter probe was incubated with the nuclear proteins and streptavidin-agarose beads to pull down DNA-bound proteins by centrifugation. *hTERT* promoter-binding proteins were separated by 12% SDS-PAGE and stained with Comassie brilliant blue. By comparing the gel lanes, significantly discrepant sections were identified, cut horizontally, and digested. The peptide mixture was extracted and analyzed by using an MDLC system coupled with a Thermo Finnigan 2-D linear ion trap mass spectrometer. By peptide mapping using an internet-based proteomics tool, we identified the candidate tumor-specific *hTERT* promoter-binding protein as a transcription elongation factor, SPT5. The SDS-PAGE image shows the *hTERT* promoter-binding proteins. The arrow indicates the tumor cell-selective *hTERT* promoter-binding protein.

The streptavidin-agarose bead pull-down assay is a reliable approach for the detection of DNA-binding proteins such as transactivators, coactivators, and mediators. We designed and synthesized a 438-bp 5′-biotinylated double-stranded oligonucleotide DNA probe corresponding to the 5′-flanking sequence of the *hTERT* gene from −378 to +60. Nuclear proteins were extracted from a normal colon epithelial cell line (CCD 841 CoN) and colon cancer cell line (RKO), respectively. The *hTERT* promoter probe was incubated with nuclear proteins and streptavidin-agarose beads to pull down DNA-bound proteins by centrifugation, which is mainly based on the high binding affinity of biotinylated DNA to streptavidin-agarose beads. *hTERT* promoter-binding proteins were separated by 12% SDS-PAGE and stained with Comassie brilliant blue, as shown in Figure 1. 5 pairs of discrepant sections were cut out and digested. The peptide mixture was extracted and analyzed using an MDLC system coupled with a Thermo Finnigan 2-D linear ion trap mass spectrometer. By peptide mapping using internet-based proteomics tools, we identified one candidate tumor-specific *hTERT* promoter-binding protein is transcription elongation factor *SPT5, which is a* protein encoded by the *SUPT5H* gene.

### Validation of SPT5 as a tumor-specific *hTERT* promoter-binding protein

**Figure 2:**
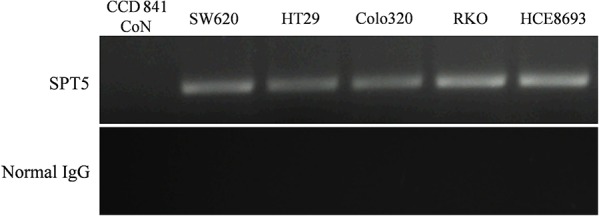
Validation of SPT5 as a tumor-specific hTERT promoter-binding protein ChIP assay was used to verify specific binding of SPT5 protein to the *hTERT* promoter. A specific antibody against SPT5 was used to precipitate chromatin, and normal IgG was used as negative control. The ChIP assay generated significant DNA bands in the SPT5-antibody immunoprecipitated samples of various colon cancer cell lines (SW620, HT29, Colo320, RKO, and HCE8693). No DNA bands were observed in the normal colon epithelial cell line (CCD 841 CoN).

ChIP is generally employed to determine specific interactions between a particular protein and DNA *in vivo*. We adopted this technique to verify the specific binding of the SPT5 protein to the *hTERT* promoter. A specific antibody against SPT5 was used to precipitate chromatin and normal IgG was used as negative control. After PCR amplification, the products were separated by gel electrophoresis. ChIP analysis showed distinct DNA bands in the SPT5-antibody immunoprecipitated samples of the colon cancer cell lines, SW620, HT29, Colo320, RKO, and HCE8693, while no DNA bands were observed in normal colon epithelial cell line, CCD 841 CoN (Figure 2). These results indicate that the SPT5 protein directly and specifically binds to the *hTERT* promoter region in colon cancer cell lines.

### *SUPT5H* and *hTERT* expression in human colon cancer cell lines

**Figure 3:**
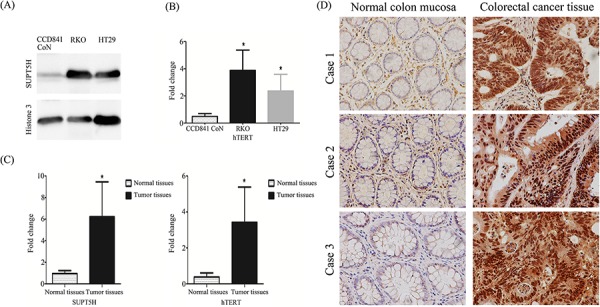
Expression of SUPT5H mRNA and protein levels and hTERT mRNA in human colorectal cancer **A.** Western blot was used to detect the expression of SPT5 protein in the nucleus of human colon cancer cell lines (RKO, HT29) and normal colon epithelial cell line (CCD 841 CoN). The results show that SUPT5H protein highly expressed in colon cancer cell lines, but almost silenced in normal colon epithelial cell line. **B.** hTERT mRNA was also highly expressed in these colon cancer cell lines compared with the normal colon epithelial cell line. **C.** The level of SUPT5H and hTERT mRNAexpression in 100 human colorectal cancer tissues was significantly higher than that in case-matched normal tissues. The measurements represent the means ± SD of triplicate experiments. *p < 0.05. **D.** Representative immunohistochemistry results show that SPT5 protein was highly expressed in the nucleus and cytoplasm of primary colorectal cancer tissues compared with that in case-matched normal tissues. Magnification, × 400.

*SUPT5H* was highly expressed in the nuclei of human colon cancer cell lines, RKO and HT29, but nearly silenced in the normal colon epithelial cell line, CCD 841 CoN (Figure 3A). Meanwhile *hTERT* mRNA was also highly expressed in these colon cancer cell lines compared with the normal colon epithelial cell line (Figure 3B).

### Positive correlation between *SUPT5H* and *hTERT* mRNA expression in 150 cases of colorectal cancer tissues

Real-time RT-PCR was used to compare the expression level of *SUPT5H* and *hTERT* mRNA between colorectal cancer tissues and case-matched normal colorectal tissues of 150 primary colorectal cancer patients. The results showed that *SUPT5H* and *hTERT* mRNA expression levels in cancer tissues were both significantly higher than those in case-matched normal colorectal tissues (*p* < 0.05; Figure 3C). Furthermore, as shown in Table 1, the results of the chi-square test and Spearman's correlation (rho = 0.726) indicated a statistically significant correlation between the levels of mRNA expression of *SUPT5H* and *hTERT* (*p* < 0.05).

Table 1The correlation between *SUPT5H* and hTERT mRNA expression in 150 cases of colorectal cancer tissues

**(A):** 

	hTERT expression			P-value
	Low	High	Total	
*SUPT5H* expression				
Low	23	10	33	P < 0.05*
High	16	101	117	
Total	39	111	150	

* Statistically significant (*P* < 0.05).

**(B):** 

	Spearman’s rho	*SUPT5H* expression	hTERT expression
*SUPT5H* expression			
	Correlation Coefficient	1.000	0.726
	Sig.(2-tailed)		P < 0.001*
	N	150	150
hTERT expression			
	Correlation Coefficient	0.726	150
	Sig.(2-tailed)	P < 0.001*	
	N	150	150

* Statistically significant (*P* < 0.01).

### Correlation between *SUPT5H* protein expression and clinicopathological features in 100 primary colorectal cancer patients

We further investigated *SUPT5H* protein expression in 10 cases of normal colon mucosa tissues and 100 cases of colorectal cancer tissues by immunohistochemistry. *SUPT5H* was expressed at a much higher level in the nucleus and cytoplasm of colorectal cancer tissues than in normal colon mucosa tissues (Figure 3D). Table 2 shows that the high level of expression of *SUPT5H* in colorectal cancer cases was significantly correlated with distant metastasis (*p* < 0.05).

**Table 2:** The correlation between *SUPT5H* protein expression and clinicopathological features in 100 cases of colorectal cancer patients

	N	SUPT5H immunoreactivity		P-value
		Low(%)	High(%)	
Total	100	44(44)	56(56)	
Gender				
Male	57	27(47.4)	30(52.6)	0.4346
Female	43	17(39.5)	26(60.5)	
Age				
≥64	50	20(40)	30(60)	0.4203
<64	50	24(48)	26(52)	
Histopathological Grading				
Well/moderately	88	41(46.6)	47(53.4)	0.2191
Poorly	12	3(25)	9(75)	
pT categories				
pT1	1	0(0)	1(100)	0.1563
pT2	20	13(65)	7(35)	
pT3	49	20(40.8)	29(59.2)	
pT4	30	11(36.7)	19(63.3)	
pN categories				
pN0	44	23(52.3)	21(47.7)	0.1396
pN1/2	56	21(37.5)	35(62.5)	
pM categories				
pM0	87	42(48.3)	45(51.7)	0.0259*
pM1	13	2(15.4)	11(84.6)	
Stage				
I/II	38	20(52.6)	18.(47.4)	0.1734
III/IV	62	24(38.7)	38(61.3)	

* Statistically significant (*P* < 0.05).

### Inhibition of SPT5 expression by shRNA in RKO and HT29 cells

**Figure 4:**
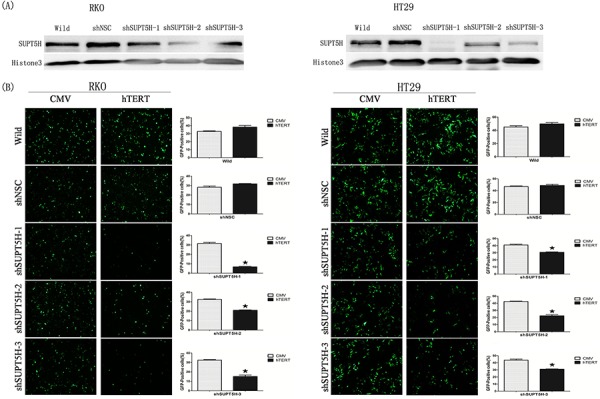
Inhibition of hTERT promoter-driven green fluorescent protein (GFP) expression by SUPT5H-specfic shRNA **A.** Western blot was used to comfirm the effectiveness of inhibition in cells. Three clones of RKO (shSUPT5H-1, shSUPT5H-2, shSUPT5H-3) and three clones of HT29 (shSUPT5H-1, shSUPT5H-2, shSUPT5H-3), which have lower expression level of SUPT5H protein than negative control (RKO-shSNC, HT29-shSNC) and wild-type cells (RKO, HT29), were used in further study. **B.** Inhibition of *SUPT5H* expression by *SUPT5H*-specific shRNA attenuated hTERT promoter-driven GFP expression in RKO (shSUPT5H-1, shSUPT5H-2, shSUPT5H-3) and HT29 (shSUPT5H-1, shSUPT5H-2, shSUPT5H-3) cells transfected with hTERT promoter-driven GFP vector, while had no obvious effect on CMV promoter-driven GFP expression. The measurements represent the means ± SD of three independent experiments. *p < 0.05.

The expression of SPT5 protein in colon cancer cells (RKO, HT29) was suppressed by *SUPT5H*-specific shRNA. Western blot analysis was employed to confirm the effectiveness of inhibition. RKO-shSNC and HT29-shSNC were used as negative controls. Three clones of RKO (shSUPT5H-1, shSUPT5H-2, and shSUPT5H-3) and three clones of HT29 (shSUPT5H-1, shSUPT5H-2, and shSUPT5H-3), which had lower expression levels of the SPT5 protein in the nuclei than that observed in the negative control and wild-type cells, were adopted for further investigation (Figure 4A).

### Inhibition of *hTERT* promoter-driven green fluorescent protein (GFP) expression by *SUPT5H*-specfic shRNA

To investigate the role of SPT5 protein in the regulation of *hTERT* promoter-driven GFP expression, *SUPT5H*-specific shRNA transfected cells, negative control cells, and wild-type cells were adopted, as previously mentioned. The results showed that inhibition of SPT5 expression by *SUPT5H*-specific shRNA attenuated *hTERT* promoter-driven GFP expression in RKO and HT29 cells transfected with *hTERT* promoter-driven GFP vector, whereas no obvious effect on CMV promoter-driven GFP expression was observed (Figures 4B). Representative graphs of fluorescence-activated cell sorting (FACS) were shown in Supplemental Figure 1. These results indicated that SPT5 is essential for *hTERT* promoter activity and *SUPT5H* transcriptionally activated *hTERT* promoter-driven gene expression in human colon cancer cells.

### The effects of *SUPT5H* protein expression on telomerase activity and telomere length

**Figure 5:**
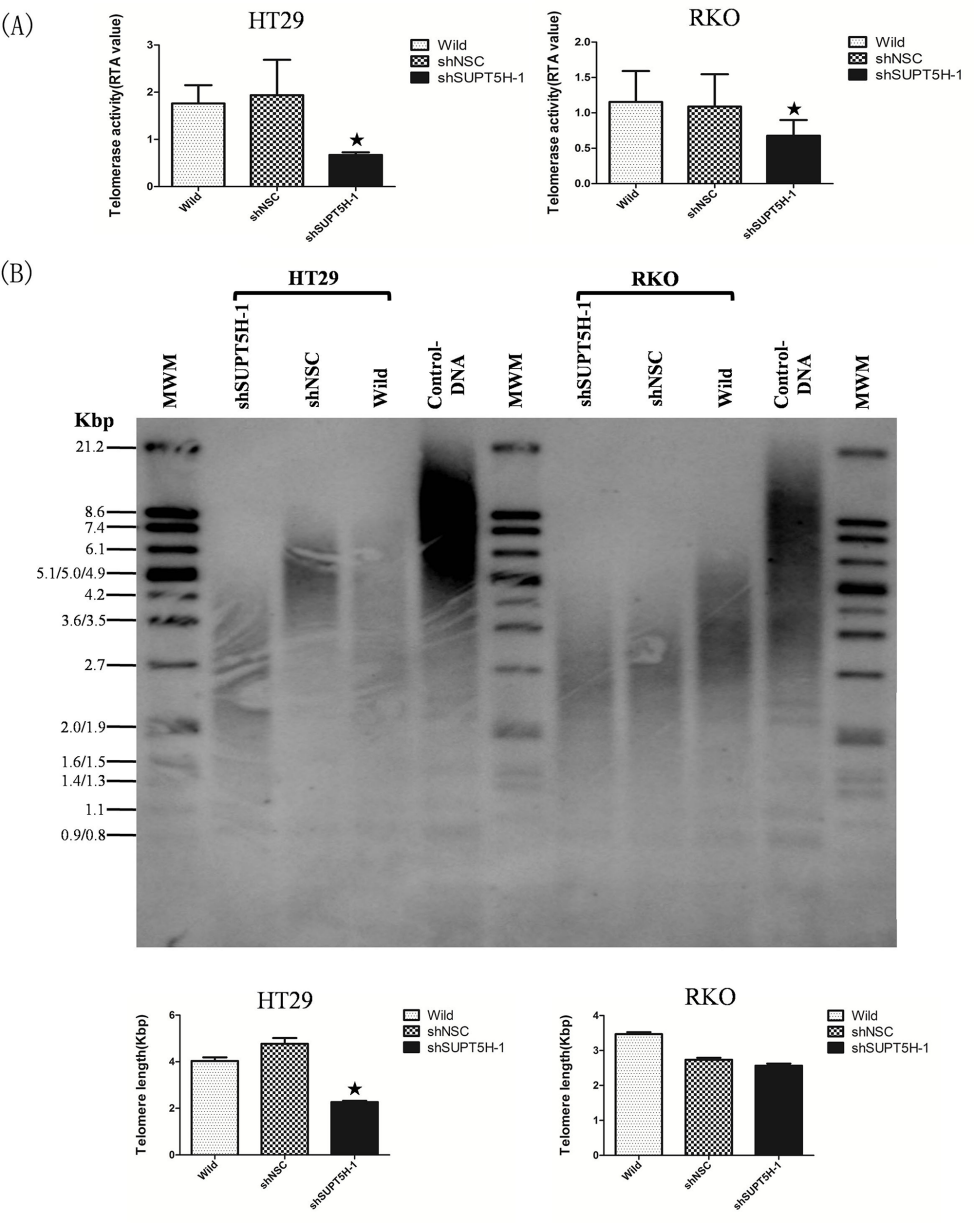
The effects of SUPT5H inhibition on telomerase activity and telomere length **A.** Significantly decreased telomerase activity was detected in HT29-shSUPT5H-1 and RKO-shSUPT5H-1. *p < 0.05. **B.** Shortened telomere length was detected in HT29-shSUPT5H-1 cells compared with negative control cells (HT29-shNSC) and wild-type cells. *p < 0.05.

We measured the telomerase activity and telomere length of wild-type cells, negative control cells (shNSC), and *SUPT5H*-specific shRNA transfected cells (shSUPT5H). A highly attenuated telomerase activity and shortened telomere length were detected in HT29-shSUPT5H-1 and RKO-shSUPT5H-1 cells treated with *SUPT5H*-specific shRNA compared to that observed in wild-type HT29, RKO and negative control cells, though the difference of shortened telomere length between RKO-shSUPT5H-1 cells and shNSC cells could not reach statistical significance (*p* < 0.05; Figures 5).

### Inhibition of *SUPT5H* expression not only promoted cell senescence in colon cancer cells, but also effectively suppressed cancer cell growth and migration

**Figure 6:**
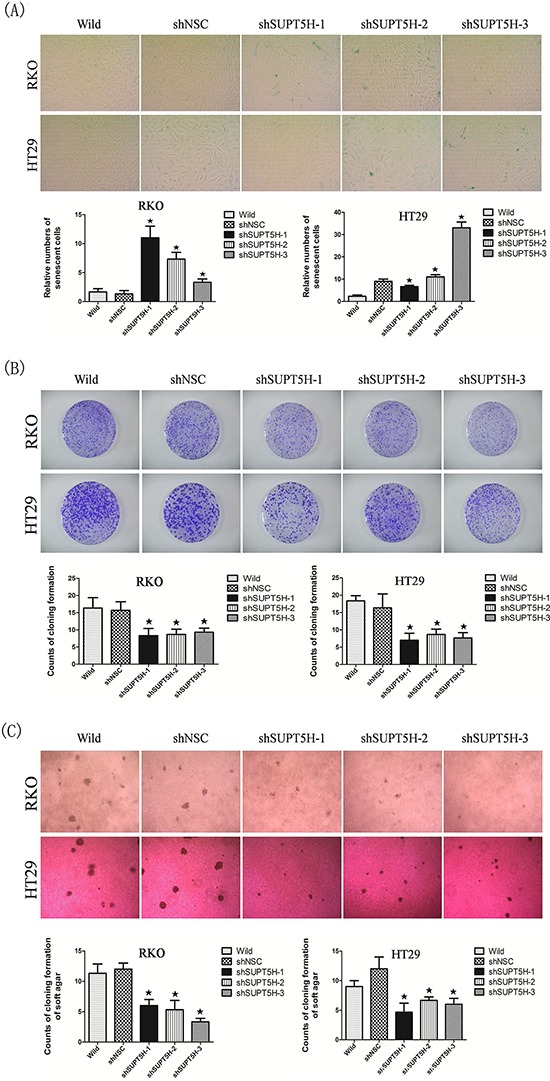
Inhibition of *SUPT5H* expression promoted cell senescence in colon cancer cells and suppressed cancer cell growth **A.** More senescent cells were observed in *SUPT5H*-knockdown cells (shSUPT5H) compared with wild-type cells or negative control cells (shNSC). *p < 0.05. (B,C) *SUPT5H* expression inhibition significantly suppressed the colony formation efficiency of cancer cells in both monolayer culture **B.** and soft agar **C.** compared to wild-type cells and negative control cells. *p < 0.05.

Increased activity for senescence-associated β-galactosidase is one of the characteristic phenotypes of senescent cells. We investigated whether suppression of *SUPT5H* expression would be sufficient to induce premature senescence by assessing the number of senescence-associated β-galactosidase-stained cells. A higher number of senescent cells was observed in *SUPT5H*-knockdown cells (shSUPT5H) compared to that observed in wild-type cells or negative control cells (shNSC) (Figure 6A). These findings indicated that suppression of *SUPT5H* expression induces senescence of colon cancer cells.

**Figure 7:**
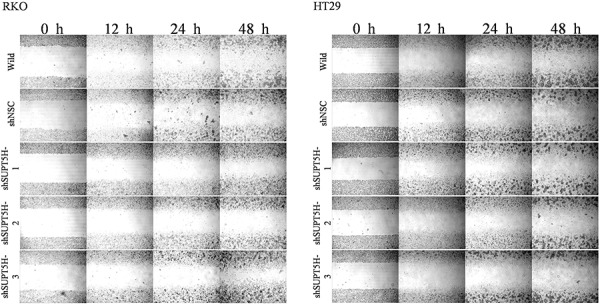
Inhibition of *SUPT5H* expression suppressed cancer cell migration Stable *SUPT5H*-suppressed clones (shSUPT5H) required a longer time (>48 h) to close a scratched wound than wild-type cells and negative control cells (shNSC).

Stable *SUPT5H*-suppressed clones (shSUPT5H) were used to investigate the effects of *SUPT5H* expression on the proliferation of colon cancer cells. Colony formation assays showed that the inhibition of *SUPT5H* expression effectively suppressed the colony formation efficiency of transfected cells in both monolayer cultures (Figure 6B) and soft agar (Figure 6C), compared to that observed in wild-type cells and negative control cells (*p* < 0.05). To determine the effects of *SUPT5H* expression on the migration of colon cancer cells, a wound-healing assay was performed. shSUPT5H cells spent a significantly longer time (>48 h) to close a scratch wound than wild-type cells and negative control cells (shNSC) (Figure 7), suggesting that *SUPT5H* promotes the proliferation of colon cancer cells.

### No effects of *SUPT5H* expression on cell cycle and apoptosis

We also determined whether suppression of *SUPT5H* expression was correlated with cell cycle arrest and apoptosis. No effects of *SUPT5H* expression on cell cycle and apoptosis were observed (**data not shown**).

## DISCUSSION

Telomerase activity has been detected in more than 85% of human tumors, whereas in normal human somatic cells, it is either undetectable or present at significantly low levels. Malignant transformation from mortal, normal cells to immortal, cancer cells is generally associated with the activation of telomerase and subsequent telomere maintenance [13]. Tumor cells do so by inducing telomerase activity through *hTERT* transcriptional upregulation and *hTERT* expression, resulting in telomerase activity. In the present study, we identified that the transcription elongation factor *SPT5, which is a* protein encoded by the *SUPT5H* gene, is a novel tumor-specific human telomerase reverse transcriptase promoter-binding protein and activator in colon cancer cells.

In all domains of life, elongating RNA polymerases require the assistance of accessory factors to maintain and regulate their rates of processivity. Among these elongation factors, SPT5 is of particular interest because it is the only RNA pol II-associated factor known to be conserved in all three kingdoms of life (NusG in bacteria, RpoE in Archaea) [14]. In Archaea and eukaryotes, SPT5 associates with a second protein, SPT4. In addition to regulating elongation, the eukaryotic SPT4-SPT5 complex appears to couple chromatin modification states and RNA processing to transcription elongation [15].

The present study determined that *SUPT5H* expression was apparently up-regulated in human colorectal cancer cell lines and primary cancer tissues. *SUPT5H* mRNA expression was positively correlated with *hTERT* mRNA expression, and *SUPT5H* protein expression was correlated with frequent distant metastasis in colorectal cancer patients. Although no report has described *SUPT5H* overexpression in colorectal cancer and other types of cancer, a previous study has demonstrated that *SUPT5H* is essential for the growth and proliferation of HeLa cells, and *SUPT5H* knockdown causes senescence [16]. Recently, SPT5 was also validated as a novel modifier of ERa protein levels in breast cancer cells. In addition, ERa intensity decreased with *SUPT5H* knockdown [17]. SPT5 was also confirmed as a relevant target of *BRCA1* interaction in breast cancer. The cancer-associated BRCT domain defects in *BRCA1* that suppressed the phosphorylated RNA polymerase II (RNAP II) carboxy terminal domain (P-CTD) cleavage and lethality in yeast also suppressed the physical interaction of *BRCA1* with human SPT5 in breast epithelial cells. Furthermore, enhanced P-CTD cleavage was observed in both yeast and human breast cells following UV-irradiation, indicating a conserved eukaryotic damage response. Therefore, the defects in this conserved *BRCA1*-dependent RNAP II cleavage and degradation pathway may be critical for the initiation of breast and/or ovarian cancer [18].

SPT5 mainly functions as an adaptor that mediates interaction of different factors with RNAP II [19]. Recruitment of SPT4-SPT5 to polymerase is mediated by a direct interaction between the RNAP II clamp domain and the SPT5 NGN domain [20]. Similar to the RNAP II CTD, SPT5 contains a series of short repeating sequence motifs at or near its C terminus, known as C-terminal repeats (CTR). The phosphorylation of SPT5 CTR and RNAP II CTR by P-TEFb complex, which is composed of CDK9 and cyclin-T (CCNT1 or CCNT2), is critical for SPT5 to exert its positive effect on transcription elongation [21]. Phosphorylated SPT5 CTR serves as a recruitment platform for the PAF complex, which recruits the other factors involved in chromatin modification and mRNA maturation [22–24]. A core promoter region of *hTERT* gene contains several putative transcription factor binding elements, including potential binding sites for Sp1, c-Myc, AP-2, AP-4, and NF-1 [25–29], which suggests that hTERT expression is regulated by different factors in various cellular contexts. Different factors may be involved in *hTERT* promoter regulation in different tumor cell lineages. Recently, two E-boxes (CACGTG), as well as a novel element (MT-box) were also identified in the *hTERT* promoter in a number of differentiation systems [30]. However, due to lack of complete structural information, the detailed function of SPT5 in the *hTERT* transcriptional process has yet to be investigated. The future work will study that whether the known binding sites in the *SUPT5H* gene are also present in the *hTERT* promoter and how SPT4-SPT5 engages and modulates the properties of elongating RNAP II during *hTERT* transcription.

Telomere attrition contributes to genomic instability and may thereby promote the development of malignant cell transformation. Furthermore, telomerase may exert functions beyond telomere lengthening during tumorigenesis [31, 32]. hTERT has been found to take part in DNA repair and to protect cells from undergoing cell cycle arrest and/or apoptosis, thus rendering cancer cells resistance against chemotherapeutic agents and/or radiation therapy [33–35]. We hypothesized that repressing hTERT activity by virtue of suppressing the expression of *SUPT5H* may give rise to anti-tumor effect, which was confirmed by the findings of the present study. Since *SUPT5H* suppression inhibited the growth and migration colon cancer cells in addition to promote senescence, though no effects on cell cycle and apoptosis were observed. However we also believe that *SUPT5H* may play an important role in colorectal cancer development by affecting genes other than *hTERT*. Because previously SPT4-SPT5 has been implicated in transcription-coupled DNA repair [36–38], a function that is executed as part of the RNAP II elongation complex. Immunoglobulin class switch recombination and somatic hypermutation also requires mammalian SPT4-SPT5 [39,40]. Mechanistically, SPT4-SPT5 facilitates the targeting of this pathway by specifically interacting with activation-induced cytidine deaminase (AID), a key enzyme that initiates these processes, which recruits this enzyme to transcription sites where RNAP II dwells (or stalls). AID does not directly interact with RNAP II, SPT4-SPT5 most likely functions as an adaptor between AID and the RNAP II apparatus. In addition, SPT4 mutations affect chromosome segregation [41], and SPT4-SPT5 is required to restrict the spread of the histone H3 variant Cse4 away from centromeres. Interestingly, ChIP studies show that SPT4-SPT5 can be found in the vicinity of centromeres, and that this localization does not depend upon RNAP II [42].

In summary, we have established that SPT5 is a novel tumor cell-specific *hTERT* promoter-binding protein in colon cancer cells. *SUPT5H* overexpression activated *hTERT* mRNA expression. Significant up-regulation of *SUPT5H* expression was observed in human colorectal cancer tissues, and inhibition of *SUPT5H* expression imparted an anti-tumor effect on colon cancer cells. *SUPT5H* may potentially serve as a biomarker for oncogenesis and development of human colorectal cancer. Further studies on the function of *SUPT5H* and transcriptional regulation of *hTERT* could lead to a better understanding of the complex regulation of human telomerase in normal and cancer cells, as well as identify new strategies for anti-cancer therapy.

## MATERIALS AND METHODS

### Cell lines, tumor samples, and normal control tissues

Normal colon epithelial (CCD 841 CoN) cell line and colon cancer cell lines (RKO, HT29, HCE8693, SW620, Colo320) were used in the present study. CCD 841 CoN were cultured in ATCC-formulated Eagle's minimum essential medium supplemented with 10% fetal bovine serum. The colon cancer cell lines were maintained in RPMI 1640 or Dulbecco's modified Eagle's medium with 10% fetal bovine serum. All cells were incubated in a humidified incubator supplied with 5% carbon dioxide.

mRNA was extracted from a total of 150 colorectal cancer tissue samples and case-matched normal colorectal tissue samples (from the same cases) from colorectal cancer patients. The other 100 paraffin blocks of colorectal cancer tissue samples that were derived from colorectal cancer patients were prepared for immunohistochemical analysis. All participating patients have not received pre-operative chemotherapy and underwent surgery at the Sir Run Run Shaw Hospital (Hangzhou, Zhejiang, China). Ten normal colon mucosa biopsy samples obtained from the healthy volunteers were used as normal controls. The ethics committee of Sir Run Run Shaw Hospital, Zhejiang University approved this study.

### Adenoviral vector

GFP-expressing adenoviruses controlled by hTERT or CMV promoter were prepared as described elsewhere [43]. Ad/CMV-GFP was used as the vector control. Adenoviral vector amplification, purification, titration, and quality testing were performed as previously reported [44]. Particle/infectious unit ratios were 100:1.

### DNA-protein binding assay

Binding of nuclear protein extracts and *hTERT* promoter DNA was assayed by a streptavidin-agarose bead pull-down assay. Nuclear protein extracts were prepared. The biotin-labeled double-stranded oligonucleotide probes were synthesized based on the *hTERT* promoter sequences encompassing −378 to +60. Then, 1 mg of the nuclear proteins, 10 μg of the biotin-labeled DNA oligonucleotides, and 100 μL of streptavidin-agarose beads (Sigma, CA, USA) were added into an Eppendorf tube in sequence to form a mixture, which was then incubated at room temperature for 2 h with continuous shaking. The beads were then pelleted by centrifugation at 500 × g in a microcentrifuge for 1 minute and washed three times with cold phosphate-buffered saline (PBS). The bound proteins were eluted for further analysis.

### Identification of *hTERT* promoter-binding proteins

The identification of *hTERT* promoter-binding proteins was performed as described elsewhere [45]. Briefly, the *hTERT* promoter-binding proteins were separated by 12% SDS-PAGE and stained with Comassie brilliant blue. Pairs of significantly distinct bands were cut out and digested. The peptide mixture from in-gel tryptic digestions was analyzed using an MDLC system (Michrom Bioresources Inc., Auburn, CA, USA) coupled with a Thermo Finnigan 2-D linear ion trap mass spectrometer (LTQ^XL^, Thermo Inc., San Jose, CA, USA). The raw MS spectra were created by using the TurboSEQUEST program in the BioWorks 3.3 software suite against the Human International Protein Index protein sequence database (IPI.Human.v3.63.fasta). The searched peptides and proteins were validated by PeptideProphet and ProteinProphet in the Trans-Proteomic Pipeline (TPP, v.4.2) using default parameters.

### Chromatin immunoprecipitation (ChIP)

Chromatin immunoprecipitation was conducted using a ChIP Assay kit (Beyotime, catalog number P2078, Nanjing, China) according to manufacturer's instructions. Briefly, nuclear proteins were cross-linked to DNA by using 1% formaldehyde at 37°C for 10 min, and then quenched with 0.125 M glycine. The cells were washed twice in cold PBS, scraped and lysed in lysis buffer (1% SDS, 10 mM EDTA, 50 mM Tris (pH 8.1), with 1 mM phenylmethylsulfonyl fluoride) for 10 min at 4°C. The lysates were sonicated 4 times for 10 s each time (on ice, 10-s break, 40% amplitude; Sonics), and the debris was removed by centrifugation. The lysates were diluted to a ratio of 1:10 in ChIP dilution buffer [0.01% SDS, 1.1% Triton X-100, 1.2 mM EDTA, 167 mM NaCl, protease inhibitors, and 16.7 mM Tris-HCl (pH 8.1)]. Approximately 70 μL of protein A+G agarose/salmon sperm DNA was added to the remaining diluted lysate and rotated for 30 min at 4°C. The mixture was centrifuged at 1000 × g for 1 min at 4°C, and then the supernatant was transferred to a fresh centrifuge tube. Approximately 1 μg of the primary antibody (anti-SUPT5H antibody and non-immune rabbit IgG; Sigma) was added to the supernatant and rotated overnight at 4°C. Then, 60 μL of protein A+G agarose/salmon sperm DNA was added to the supernatant to allow precipitation of SUPT5H or IgG-associated genomic DNA fragments by rotating for 60 min at 4°C, followed by washing with different buffers as follows: low salt immune complex wash buffer, once; high salt immune complex wash buffer, once; LiCl immune complex wash buffer, once; and TE buffer, twice. Chromatin was extracted in an elution buffer (1% SDS and 0.1 M NaHCO3). The cross-linkages of the DNA-protein complexes were reversed at 65°C for 4 h. The DNA was finally extracted using the standard phenol-chloroform method and subjected to PCR amplification using specific *hTERT* promoter primers. (5′-primer, ^−346^GCCGATTCGACCTCTCTCC^−328^; and 3′-primer, ^+234^AAGGTGAAGGGGCAGGAC^+251^). The resulting 232-bp product of the *hTERT* promoter was separated by 1.2% agarose gel electrophoresis.

### Western blot analysis

Western blot analysis was performed with a specific antibody to SPT5 (sc-28678, Santa Cruz Biotechnology, Santa Cruz, CA, USA) or histone 3 (AH433, Beyotime, Nanjing, China). Detection was performed using an ECL kit (Pierce Chemical Co., Rockford, IL, USA), and the blots were developed using a Fujifilm Las-4000 Imaging System.

### Reverse transcription quantitative real-time polymerase chain reaction (RT-qPCR)

cDNA synthesis was performed using SuperScript II reverse transcriptase (Invitrogen) with oligo(dT) and random hexamer primers (Invitrogen), according to the manufacturer's instructions. RT-qPCR for cDNA panels of the human multiple colorectal cancer tissues containing their case-matched normal colorectal tissues cDNA panels was performed by using SsoFast^TM^ EvaGreen^®^ supermix with low ROX (Bio-Rad). GAPDH mRNA was also amplified as internal control. The sense and antisense primers used for SUPT5H were as follows: 5′-CCCAGCAGGCTACCAGAATA-3′ and 5′-ATGCCATCCTCACCATCAAT-3′, respectively. The sense and antisense primers used for *hTERT* were as follows: 5′-GCCTTCAAGAGCCACGTC-3′ and 5′-CCACGAACTGTCGCATGT-3′, respectively. The PCR conditions were as follows: a 2-min initial denaturation at 95°C, followed by 40 cycles of 95°C for 5 s and 60°C for 34 s, then 1 cycle of 95°C for 15 s, 60°C for 1 min, 95°C for 15 s and 60°C for 15 s. Tissue templates and internal controls were run in triplicate.

### Immunohistochemistry

The ChemMate EnVision Detection Kit (Dako, Carpinteria, CA, USA) and paraffin blocks of colorectal cancer tissue samples and normal colon mucosa biopsy samples were used for immunohistochemical analysis. Briefly, the paraffin-embedded samples were deparaffinized in xylene and dehydrated with ethanol. For antigen retrieval, the paraffin-embedded sections were placed in 0.01 M sodium citrate buffer (pH 6.0) and subjected to pressure cooker treatment for 5 min at full pressure. After cooling to room temperature, the sections were incubated with 3% hydrogen peroxide to block endogenous peroxidase activity. Then, nonspecific antigens were blocked with 10% goat serum (100 μL goat serum in 900 μL PBS) at room temperature for 30 min. The sections were incubated with the primary antibody (1:200 dilution) overnight at 4°C. The primary antibody used in the present study was a rabbit polyclonal antibody against the human SUPT5H protein (HPA029273, Sigma, CA, USA). Then, the ChemMate EnVision/HRP, Rabbit/Mouse (ENV) reagent was applied to the sections, followed by application of ChemMate DAB+ Chromogen, which was included in the kit. The sections were counterstained with hematoxylin for 3 min and then were rinsed, dehydrated, and covered with a glass slip.

### *SUPT5H* knockdown using RNA interference

The colon cancer cell lines, RKO and HT29, were cultured in 6-well plates, and upon reaching 50% confluency, were transfected using validated shRNA specific for SUPT5H mRNA (TG309005, OriGene, USA). The sequences of 29mer shRNA constructs in retroviral GFP vector were as follows: GI336015, CCGTTGGCTACAGTCCTATGACACCTGGA; GI33 6016, GCTTGGCTACTGGAACCAGCAGATGGTGC. A shRNA pGFP-V-RS plasmid (TR30007, shNSC) was used as negative control. Cells were transfected using MegaTran 1.0 transfection reagent (TT200002, OriGene, USA). Stable *SUPT5H*-suppressed clones were selected for further analysis.

### FACS analysis

Cells were collected and suspended in cold PBS at a density of approximately 1 × 10^6^ cells/mL, followed by analysis using a FACSCalibur^TM^ flow cytometer (BD Biosciences) and CellQuest^TM^ Pro software (BD Biosciences). The percentage of GFP-positive cells was calculated.

### Telomerase activity assay

Telomerase activity of stable *SUPT5H*-suppressed clones, negative control clones, and wild-type cells were analyzed by photometric enzyme immunoassay for quantitative determination utilizing the telomeric repeat amplification protocol with a TeloTAGGG telomerase PCR enzyme-linked immunosorbent assay^plus^ kit (12013789001, Roche Applied Science), following the manufacturer's instruction.

### Telomere length assay

Telomere length of stable *SUPT5H*-suppressed clones, negative control clones, and wild-type cells were measured by using a nonradioactive chemiluminescence assay that utilized Southern blot analysis of terminal restriction fragments obtained by digestion of genomic DNA with frequently cutting restriction enzymes (*HinfI/RsaI*) with a TeloTAGGG telomere PCR length assay kit (12209136001, Roche Applied Science), following the manufacturer's instructions. Telomeric smears were developed using a Fujifilm Las-4000 Imaging System.

### Colony formation assay

For the colony formation assays, stable *SUPT5H*-suppressed clones, negative control clones, and wild-type cells (density: 1 × 10^3^/well) were plated in 10-cm dishes, respectively. After 10 days, colonies (≥50 cells/colony) were counted after 0.1% crystal violet staining.

For the soft agar assay, ∼500 cells were suspended in a medium containing 0.3% low-melt agarose, seeded into a 6-well plate overlaid with 0.5% low-melt agarose, and allowed to grow for 2 weeks at 37°C in 5% CO_2_. Colonies containing >50 cells were counted under a microscope. Three wells were analyzed for each experiment.

### Wound-healing assay

Cell migration assay was performed using wound-healing assay. Stably transfected cells were cultured in 35-mm dishes until confluency. The cell layers were carefully wounded using a 10-μL sterile tip across each well, creating a cell-free area, and washed twice with fresh medium. Images of the same microscopic field were captured at various time points (0, 12, 24, and 48 h).

### Senescence assay

Senescence assay was performed with a senescence β-galactosidase staining kit (#9860, Cell Signaling Technology, Inc.) following the manufacturer's instructions. Sub-confluent cells were washed once with 2 mL of PBS per 35-mm dish to sufficiently cover the cells for 30 s. Approximately 1 mL of a fixative solution per 35-mm dish was added to submerge the cells and then incubated at room temperature for 15 min. The cells were washed twice with 2 mL of PBS per 35-mm dish. Approximately 1 mL of a β-galactosidase staining solution per 35-mm dish was added. The plates were incubated at 37°C overnight in a dry incubator (no CO_2_). The stained cells were observed under a microscope (200 × total magnification) to detect the development of a blue stain. The experiments were performed in triplicate.

### Cell cycle and apoptosis analyses

Cell cycle distribution and the percentage of apoptosis were determined by flow cytometry as described elsewhere [46].

### Statistical analyses

The results were expressed as the mean ± SD. Statistical analysis was performed by using SPSS 11.0 for Windows (SPSS Inc., Chicago, IL, USA). The chi-square test was used to analyze the association of *SUPT5H* expression with the different clinicopathological parameters, and Correlation coefficients between *SUPT5H* and *hTERT* mRNA expression were tested by using *Spearman*’*s rho*. *P* < 0.05 was considered statistically significant.

## SUPPLEMENTARY MATERIALS FIGURE


