# Supplementary material for: Suppressor of Ty homolog-5, a novel tumor-specific human telomerase reverse transcriptase promoter-binding protein and activator in colon cancer cells
Source: Oncotarget. 2015 Sep 25;6(32):32841–55. doi: 10.18632/oncotarget.5301 (PMC4741733; doi:10.18632/oncotarget.5301)
Supplement: Supplementary file 1 [file oncotarget-06-32841-s001.pdf]

# SUPPLEMENTARY FIGURE

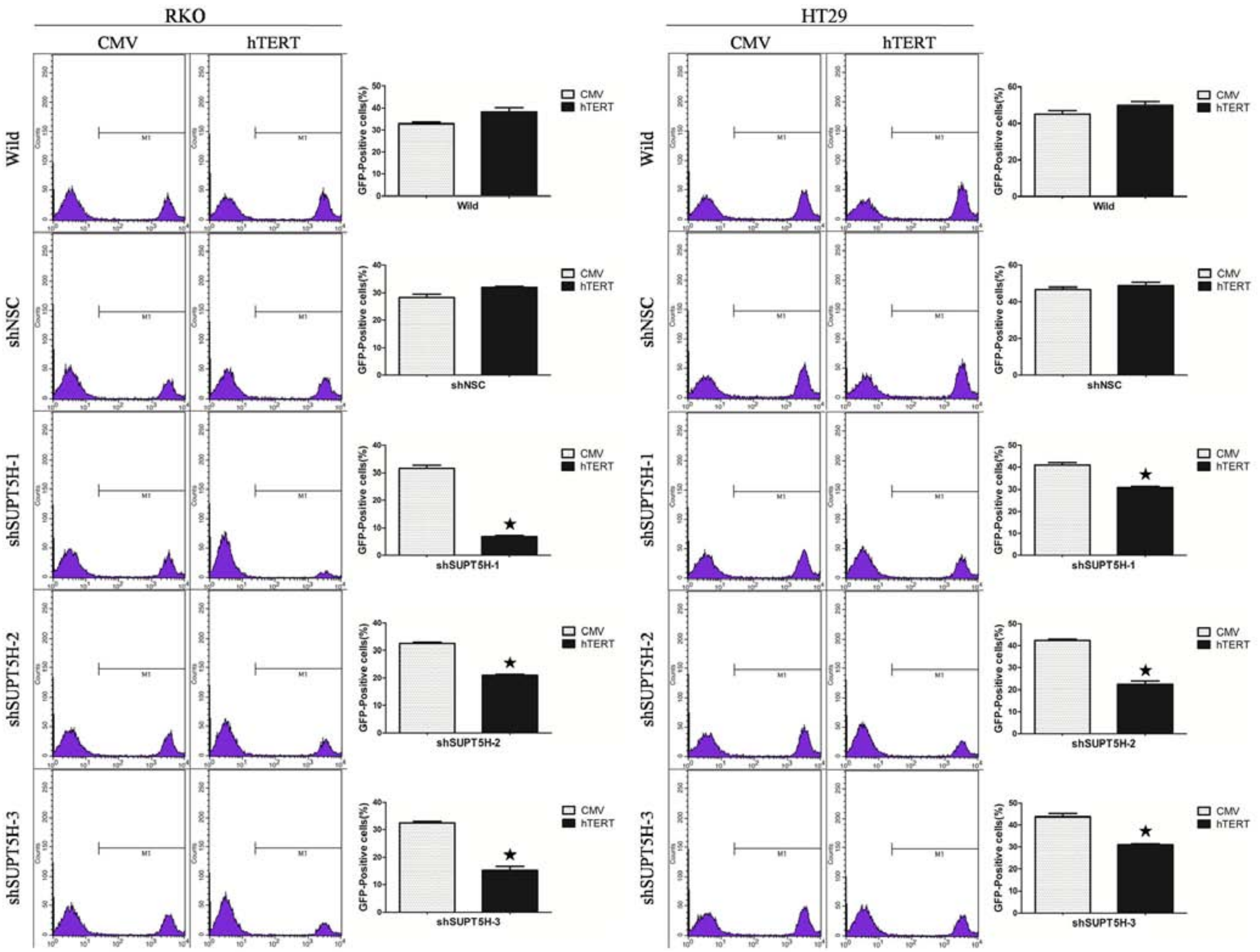


**Supplementary Figure S1: Representative graphs of fluorescence-activated cell sorting (FACS) showing the inhibition of hTERT promoter-driven green fluorescent protein (GFP) expression by SUPT5H-specfic shRNA.** The measurements were expressed as the mean ± SD of three independent experiments. $*p < 0.05$.
